# 

**Published:** 1897-10-16

**Authors:** 


					o
"CADBURY'S S?L^LV-W15' r?re CADBURY'S 0
COCOA frf JET* COCOA
'7tttSlS'--iS."' P""i'"^SEsi,? NO ALKALIES USED. .
WHICH HOSPLTAJL
No. 577. Vol. XXIII. [SSSkS Saturday,
Oct. 16th, 1897.
[Subscription Terms,"j price twopence.

				

